# The impact of synbiotic administration through in ovo technology on the microstructure of a broiler chicken small intestine tissue on the 1^st^ and 42^nd^ day of rearing

**DOI:** 10.1186/s40104-017-0193-1

**Published:** 2017-08-01

**Authors:** A. Sobolewska, J. Bogucka, A. Dankowiakowska, G. Elminowska-Wenda, K. Stadnicka, M. Bednarczyk

**Affiliations:** 1 Department of Animal Biochemistry and Biotechnology, University of Science and Technology in Bydgoszcz, Mazowiecka 28, 85-084 Bydgoszcz, Poland; 2 Department of Normal Anatomy, Ludwik Rydygier Collegium Medicum in Bydgoszcz, Nicolaus Copernicus University in Toruń, Karłowicza 24, 85-092 Bydgoszcz, Poland

**Keywords:** Broiler chicken, In ovo, Small intestine, Synbiotics

## Abstract

**Background:**

Application the innovative method which is in ovo technology provides a means of modulating the immune system at early embryonic stages. The aim of study was to determine influence of the in ovo stimulation, on d 12 of incubation, with synbiotics (synbiotic 1- *L. salivarius* IBB3154 + Bi^2^tos, Clasado Ltd. and the synbiotic 2 - *L. plantarum* IBB3036 + lupin RFOs) on the microstructure of duodenum, jejunum and ileum in the 1^st^ and 42^nd^ day of rearing.

**Results:**

On the 1^st^ day of chickens life, in the duodenum of both experimental groups (SYN1 and SYN2), a significantly higher and wider intestinal villi as well as a significantly larger absorbent surface of these villi were found in comparison with the Control group (*P* ≤ 0.01). On the 42^nd^ day of rearing the beneficial effect of synbiotic 1 was reflected by the numerically higher villi (no statistical differences) with a larger surface (*P* ≤ 0.01) in the duodenum in the SYN1 group compare to the Control group. In the jejunum on the 1^st^ day of life, in the SYN1 group, significantly higher villi than in the Control group, with a simultaneous decrease in the depth of crypts (*P* ≤ 0.01), and also the largest width of villi and their absorbent area (*P* ≤ 0.01) in comparison to the other groups were found. On the 42^nd^ day of life, in the jejunum, an increase in the height of the villi whilst reducing the crypt depth in the SYN2 group was found (*P* ≤ 0.01). In turn, in the SYN1 group, there were significantly more neutral goblet cells observed compared with the control group (*P* ≤ 0.05). In the ileum of 1-day-old chickens, the widest villi (*P* ≤ 0.05) and the deepest crypts (*P* ≤ 0.01) were found in the SYN2 group. In the same group, there was also the least amount of neutral goblet cells in comparison to the other groups (*P* ≤ 0.05).

**Conclusions:**

We observed that synbiotic 1 and 2 beneficially affected the examined characteristics on the 1^st^ and 42^nd^ day of life. The obtained results allow us to conclude that the use of synbiotics significantly affect gut structure which should contribute to improvement in nutrient absorption by the gut.

## Background

Synbiotics consist of a combination of synergistically interacting probiotics and prebiotics which can be aimed at improving the resistance and stability of health-promoting organisms in the gut of birds by providing a substrate for fermentation [1, 2]. Thus, synbiotics are factors that modulate the immune system of birds by acting on the bacterial flora of their gastrointestinal tract [3]. To ensure the greatest protection of the immune system for newly hatched chickens, external supplementation with bioactive substances, such as synbiotics, should occur as early as possible. Application of an innovative method, such as in ovo technology, provides a means of modulating the immune system at early embryonic stages. This technology involves the introduction - on the appropriate day of embryonic development of birds - of the particular substance in solution form to the air chamber of eggs or directly into a developing embryo [4]. Of course, the effectiveness of the injection and the level of use of the injected bioactive substance by the avian embryo depend on various factors [5]. These can be the chemical and physical features of the injected substances, its dose and the egg surface where the injection was performed (i.e. the embryo, the amnion, the allantois, the air chamber egg or the yolk sac). A thorough understanding of the various stages of embryonic development in birds allows the optimal time of injection to be defined [6, 7]. According to Villaluenga et al. [8], the optimal time of prebiotic injection is on the 12^th^ day of embryonic development. In comparison with injections on d 1, 8 and 17, a significantly higher number of Bifidobacteria was observed in the colon of two-day-old chickens. A similar result was obtained by Pilarski et al. [9], who studied the effect of alpha-galactosides (RFOs) administered on the 12^th^ day of egg incubation on selected traits of chickens.

This is mainly due to the fact that on this day of bird embryonic development, allantochorion is already fully developed and vascularised. The embryo is surrounded by the amniotic fluid that remains in contact with the embryonic gastrointestinal tract, which permits the transport of substances from the air chamber into the intestine [10]. Thus, a highly vascularised allantochorion enables efficient transport of bioactive substances given by injection in ovo on the 12^th^ day of egg incubation between the air chamber of the eggs and the digestive tract of chickens. Upon hatching, the in ovo modulated profile of the gut microflora has an influence on the good condition of health of a chick, eliminating the need for antibiotics. We presume that this beneficial condition of the GI (gastrointestinal) tract would be reflected in the morphology of the intestines and might be maintained throughout the life of the chicken.

The prebiotics used in this study as components of the synbiotics are: commercially developed non-digestive transgalacto - oligosaccharides (Bi^2^tos, Clasado Ltd.) and raffinose family oligosaccharides (RFOs), which was obtained from lupin seeds as a white powder.

In previous projects carried out by our team, we evaluated the impact of bioactive substances in the form of pre-, pro- and synbiotics injected on the 12^th^ day of incubation into the air chamber of the egg on production traits and the macro- and microstructure of the small intestine. The best composition of these substances and their optimal dose was selected. On the basis of these studies, we found the most beneficial effect of synbiotics on the above-mentioned parameters [11, 12].

The aim of study was to determine the influence of the in ovo stimulation with synbiotics on the microstructure of the duodenum, jejunum and ileum on the 1^st^ and 42^nd^ day of rearing.

## Methods

The experiment was conducted on hatching eggs of the Cobb 500 FF line incubated in commercial hatchery conditions (Drobex - Agro Ltd., Solec Kujawski, Poland) in Petersime incubators and on 1- and 42-day-old broilers. On d 12 of incubation, the eggs were candled, and the infertile ones or those containing dead embryos were discarded. Eggs containing living embryos were randomly divided into 3 groups. Bioactive substances were administered in an amount of 0.2 mL into the air chamber of the egg on the 12^th^ day of embryonic development by the in ovo technique. The hole in the shell of the egg was sealed with the use of a special automatic system [13]. 5000 eggs were injected. The SYN1 group received synbiotic *L. salivarius* IBB3154 + Bi^2^tos, Clasado Ltd. (2 mg of Bi^2^tos prebiotic +10^5^ bacteria/egg), and the SYN2 group received symbiotic *L. plantarum* IBB3036 + lupin RFOs (2 mg of RFO prebiotic +10^5^ bacteria/egg). The Control group was injected with physiological saline 0.9% NaCl.

### Animals

The rearing experiment was conducted on the experimental farm of the PIAST company in Olszowa according to the technological recommendations and lasted for 42 d. All groups were fed and watered ad libitum*.* Commercial diets were used according to the age of the chickens: 1-10 d - starter, 10–20 d - grower, 20-41 d – finisher (Table 1).

**Table 1:** Composition of premix for starter, grower and finisher diets for chickens

		Inclusion rate	kg/t feed	Starter diet		Grower diet		Finisher diet	
Vitamin/Element	Declared name	Additive Code	Units	Quantity per tonne of finished feed	Quantity of compound per tonne of finished feed	Quantity per tonne of finished feed	Quantity of compound per tonne of finished feed	Quantity per tonne of finished feed	Quantity of compound per tonne of finished feed
Vitamin A	Retinyl acetate	E672	MIU	13.00	-	10.00	-	10.00	-
Vitamin D_3_	Cholecalciferol	E671	MIU	5.00	-	5.00	-	5.00	-
Vitamin E	alpha Tocopherol	3a700	g	80.00	-	50.00	-	50.00	-
Vitamin K	Vitamin K	-	g	3.00	-	3.00	-	3.00	-
Vitamin B_1_	Vitamin B_1_	-	g	3.00	-	2.00	-	2.00	-
Vitamin B_2_	Vitamin B_2_	-	g	9.00	-	8.00	-	6.00	-
Vitamin B_6_	Vitamin B_6_	3a831	g	4.00	-	3.00	-	3.00	-
Vitamin B_12_	Vitamin B_12_	-	mg	20.00	-	15.00	-	15.00	-
Biotin	Biotin	-	g	0.15	-	0.12	-	0.12	-
Cal-D-Pan	Calcium Pantothenate	-	g	15.00	-	12.00	-	10.00	-
Nicotinic acid	Nicotinic acid	-	g	60.00	-	50.00	-	50.00	-
Folic	Folic acid	-	g	2.00	-	2.00	-	1.50	-
Choline	Choline chloride	-	g	0.50	-	0.40	-	0.35	-
Methio-nine	Methionine	3.1.1	g	3405.00	-	3018.00	-	2514.00	-
Threonine	Threonine	3.3.1	g	745.00	-	726.00	-	361.00	-
Lysine	Lysine	3.2.3	g	2812.00	-	2831.00	-	1779.00	-
Iodine	Calcium iodate	E2	g	1.00	1.59	1.00	1.59	1.00	1.59
Selenium	Sodium selenite	E8	g	0.35	7.78	0.35	7.78	0.35	7.78
Iron	Ferrous sulphate	E1	g	40.00	133.33	40.00	133.33	40.00	133.33
Molybdenum	Sodium molybdate	E7	g	0.50	1.27	0.50	1.27	0.50	1.27
Manganese	Manganous oxide	E5	g	100.00	161.29	100.00	161.29	100.00	161.29
Copper	Cupric sulphate	E4	g	15.00	60.00	15.00	60.00	15.00	60.00
Zinc	Zinc oxide	E6	g	100.00	138.89	100.00	138.89	100.00	138.89

### Histomorphological examination

The material for the morphological and histological assays (approx. 2 cm) of the small intestine (duodenum, jejunum, ileum) were collected from 1- and 42-day-old chickens. Before slaughter, 680 chickens from each group were weighed, and their mean body weight was calculated. Subsequently, 15 birds per group, with a body weight similar to the mean for the group, were selected. Directly after slaughter, the small intestine was extracted, and all sections of the small intestine was excised, measured and weighed. Samples were taken from the midpoint of the duodenum, from the midpoint of the jejunum between the point of entry of the bile duct and Meckel’s diverticulum and the midpoint of the ileum between Meckel’s diverticulum and the ileocecal junction. A total of 270 samples were collected (3 groups × 3 sections × 15 birds × 2 repetitions). The individual sections of the intestine were flushed with 0.9% saline and then fixed in 4% CaCO_3_ buffered formalin. The fixed samples were dehydrated, cleared and permeated with paraffin in a tissue processor (Thermo Shandon, Chadwick Road, Astmoor, Runcorn, Cheshire, United Kingdom), and subsequently embedded in paraffin blocks using an embedding system (Medite, Burgdorf, Germany). Thus, the formed blocks were cut into 10 μm-thick sections using a rotary microtome (Thermo Shandon, Chadwick Road, Astmoor, Runcorn, Cheshire, United Kingdom), which in turn were placed on microscope slides coated with egg protein with the addition of glycerine.

Before staining, the preparations were deparaffinised and hydrated. They were subsequently subjected to PAS (Periodic acid–Schiff) staining with Schiff’s reagent to conduct morphometric analyses of the small intestine and to stain and count neutral goblet cells. Measurements were made using a DELTA EVOLUTION 300 microscope equipped with a digital camera by ToupCam™. Measurements included: height and width of intestinal villi and intestinal crypt depth, and the number of neutral goblet cells in the intestine were calculated using the MultiScanBase v. 18.03 (Computer Scanning Systems II, Warsaw, Poland). In order to measure the height of the villi, ten villi per bird were randomly selected from a cross section. The length was measured from the tip of the villus to its base at the crypt-villus outlet. The perimeter length of the villus was measured to calculate the surface area using the formula cited by Sakamoto et al. [14]. The depth of intestinal crypts was defined as the invagination depth between neighbouring intestinal villi and was measured between 10 villi [15]. The number of PAS - positive cells was calculated per 1 mm^2^ of the intestinal villi surface area.

The data were analysed by means of a one-way analysis of variance using STATISTICA 10.0 PL. The arithmetic mean ($\overline{\times}$) and the standard error of the mean (SEM) were calculated. Significant differences between the groups were tested using Duncan’s Multiple Range Test.

## Results

Synbiotic *L. plantarum* IBB3036 + lupin RFO (SYN2 group) significantly increased the body weight of chickens on the 1^st^ day of life (*P* ≤ 0.05) (Table 2), while significantly higher feed intake was found in both injected groups compared to the Control group (*P* ≤ 0.05) (Table 3). FCE (Feed Conversion Ratio) did not significantly increase after synbiotics throughout the entire rearing period (Table 3). In all study groups, we also observed a very low mortality rate, which was below 2% [16].

**Table 2:** Effect of synbiotics treatment injected in ovo on d 12 of incubation on the body weight of broiler chickens

Item	Day	
	1	42
Body weight, g		
Control	40.7^b^	3127
SYN1	40.3^b^	3146
SYN2	41.6^a^	3111
SEM	0.1803	9.7890

^a-b^Difference (*P* ≤ 0.05) between treatments (vertical), Means ± SEM representing 680 birds per group. SYN 1 - *L. salivarius* IBB3154 + Bi^2^tos, Clasado Ltd., SYN 2 - *L. plantarum* IBB3036 + lupin RFOs

**Table 3:** Effect of synbiotics treatment injected in ovo on d 12 of incubation on the feed intake and on the FCE (the efficiency of feed conversion) of broiler chickens

Item	Day	
	1-10	1-41
Feed intake, g		
Control	247^b^	4930
SYN1	254^a^	4940
SYN2	258^a^	4898
SEM	1.3291	24.4097
FCE, g/g		
Control	1.21	1.60
SYN1	1.24	1.59
SYN2	1.29	1.60
SEM	0.0146	0.0067

^a-b^Difference (*P* ≤ 0.05) between treatments (vertical), Means ± SEM representing 680 birds per group. SYN 1 - *L. salivarius* IBB3154 + Bi^2^tos, Clasado Ltd.,SYN 2 - *L. plantarum* IBB3036 + lupin RFOs

While macroscopically evaluating the small intestines of the chickens, we found that the use of synbiotic 2 reduced the length (*P* ≤ 0.05 and *P* ≤ 0.01) and weight (*P* ≤ 0.01) of the duodenum on the 1^st^ day of life of the chickens in relation to the other groups (Table 4). However, it did not affect the microstructure of the analysed section at this age, because in the duodenum, in both experimental groups (SYN1 and SYN2), a significantly higher and wider intestinal villi as well as a significantly larger absorbent surface of these villi were found in comparison with the Control group (*P* ≤ 0.01) (Table 5). Simultaneously, in the SYN1 group,a significant decrease in the depth of crypts relative to the SYN2 group was reported. In the same group, a reduction in the number of goblet cells on the surface of 1 mm^2^ of the villi was observed, while the villi were significantly larger compared to the Control group (*P* ≤ 0.01) (Table 5). In turn, on the 42^nd^ day of rearing after treatment of synbiotic 1 (SYN 1 group),a greater weight of the duodenum (*P* ≤ 0.01) than in the other groups and an increased length of this section (*P* ≤ 0.05) compare to the Control group were observed (Table 4). This beneficial effect was reflected by the numerically higher villi (no statistical differences) with a larger surface (*P* ≤ 0.01) in the duodenum in the SYN1 group compare to the Control group (Table 5).

**Table 4:** Effect of synbiotics treatment injected in ovo on d 12 of incubation on the length and on the weight of intestine of broiler chickens

Item			Day	
			1	42
Length of intestine, cm	Duodenum	Control	9.60 ± 0.24^Aa^	28.67 ± 0.73^b^
		SYN 1	9.03 ± 0.24^a^	31.37 ± 0.59^a^
		SYN 2	8.28 ± 0.19^Bb^	30.10 ± 0.96^ab^
	Jejunum	Control	17.20 ± 0.65	64.00 ± 1.93^b^
		SYN 1	16.60 ± 0.65	70.20 ± 2.34^a^
		SYN 2	17.27 ± 0.49	68.50 ± 1.86^ab^
	Ileum	Control	15.60 ± 0.41	60.80 ± 1.73^b^
		SYN 1	14.77 ± 0.55	68.30 ± 2.03^a^
		SYN 2	14.54 ± 0.47	66.47 ± 2.15^a^
Weight of intestine, g	Duodenum	Control	0.44 ± 0.01^a^	17.44 ± 0.36^B^
		SYN 1	0.41 ± 0.01^ab^	19.38 ± 0.39^A^
		SYN 2	0.38 ± 0.02^b^	17.22 ± 0.45^B^
	Jejunum	Control	0.47 ± 0.03	45.31 ± 1.39^AB^
		SYN 1	0.43 ± 0.02	48.12 ± 2.19^A^
		SYN 2	0.44 ± 0.01	40.61 ± 1.72^B^
	Ileum	Control	0.36 ± 0.02	39.25 ± 1.37
		SYN 1	0.32 ± 0.02	41.45 ± 2.65
		SYN 2	0.32 ± 0.01	35.98 ± 1.86

^a-b^Difference (*P* ≤ 0.05), ^A-B^difference (*P* ≤ *0.01*) between treatments (vertical), Means ± SEM representing 15 birds. SYN 1 - *L. salivarius* IBB3154 + Bi^2^tos, Clasado Ltd., SYN 2 - *L. plantarum* IBB3036 + lupin RFOs

**Table 5:** Effect of synbiotics injected in ovo on the duodenum morphology of chickens at 1^st^ and 42^nd^ day of age

Item	Day	
	1	42
Villus height, μm		
Control	529.96 ± 19.96^B^	1889.79 ± 39.79
SYN1	603.47 ± 12.39^A^	1940.95 ± 36.10
SYN2	594.37 ± 17.07^A^	1880.52 ± 30.88
Villus width, μm		
Control	62.88 ± 1.62^B^	188.59 ± 5.13^B^
SYN1	75.72 ± 1.23^A^	216.69 ± 8.05^A^
SYN2	73.96 ± 1.71^A^	202.13 ± 5.45^AB^
Villus surface area, μm^2^		
Control	106,267 ± 6158.5^B^	1123,441 ± 39,725.1^B^
SYN1	144,041 ± 4238.0^A^	1323,359 ± 58,298.1^A^
SYN2	139,322 ± 5970.8^A^	1187,036 ± 32,976.4^AB^
Crypt depth, μm		
Control	49.95 ± 2.13^AB^	136.82 ± 3.54^B^
SYN1	45.81 ± 1.89^B^	189.31 ± 4.26^A^
SYN2	53.97 ± 1.27^A^	138.47 ± 2.30^B^
No. of neutral goblet cells/1 mm^2^		
Control	808 ± 49.08^A^	134 ± 8.78
SYN1	526 ± 21.30^B^	116 ± 4.85
SYN2	456 ± 14.28^C^	133 ± 3.63

^A-C^Difference (*P* ≤ 0.01) between treatments (vertical), Means ± SEM representing 15 birds. SYN 1 - *L. salivarius* IBB3154 + Bi^2^tos, Clasado Ltd., SYN 2 - *L. plantarum* IBB3036 + lupin RFOs

In the jejunum on the 1^st^ day of life, similar to the duodenum, in the SYN1 group, significantly higher villi than in the Control group, with a simultaneous decrease in the depth of crypts (*P* ≤ 0.01), and also the largest width of villi and their absorbent area (*P* ≤ 0.01) in comparison to the other groups were found (Table 6). On the 42^nd^ day of life, in the jejunum, an increase of the villi height whilst reducing the crypt depth in the SYN2 group was found (*P* ≤ 0.01). In turn, in the SYN1 group, there were significantly more neutral goblet cells observed compared with the control group (*P* ≤ 0.05), (Table 6).

**Table 6:** Effect of synbiotics injected in ovo on the jejunum morphology of chickens at 1^st^ and 42^nd^ d of age

Item	Day	
	1	42
Villus height, μm		
Control	337.88 ± 11.01^B^	1334.68 ± 46.19^AB^
SYN1	383.86 ± 10.01^A^	1190.20 ± 50.03^B^
SYN2	352.47 ± 10.80^AB^	1517.95 ± 54.52^A^
Villus width, μm		
Control	51.64 ± 1.06^b^	170.44 ± 7.44
SYN1	56.42 ± 1.30^a^	178.89 ± 8.10
SYN2	52.55 ± 1.46^b^	172.66 ± 5.66
Villus surface area, μm^2^		
Control	55,022.6 ± 2410.6^B^	724,927.0 ± 50,976.2
SYN1	67,746.0 ± 2309.5^A^	688,644.6 ± 51,191.4
SYN2	58,459.9 ± 2489.5^B^	814,432.8 ± 30,911.1
Crypt depth, μm		
Control	46.92 ± 0.85^A^	150.87 ± 7.65^A^
SYN1	37.59 ± 0.93^B^	125.41 ± 2.87^B^
SYN2	48.00 ± 0.97^A^	112.88 ± 3.95^B^
No. of neutral goblet cells/1mm^2^		
Control	743 ± 42.21	153 ± 20.30^b^
SYN1	778 ± 32.57	232 ± 26.24^a^
SYN2	681 ± 22.49	185 ± 6.49^ab^

^a-b^Difference (*P* ≤ 0.05), ^A-B^difference (*P* ≤ 0.01) between treatments (vertical), Means ± SEM representing 15 birds. SYN 1 - *L. salivarius* IBB3154 + Bi^2^tos, Clasado Ltd., SYN 2 - *L. plantarum* IBB3036 + lupin RFOs

In the ileum of 1-day-old chickens, the widest villi (*P* ≤ 0.05) and the deepest crypts (*P* ≤ 0.01) were found in the SYN2 group. In the same group, there was also the least amount of neutral goblet cells in comparison to the other groups (*P* ≤ 0.05) (Table 7). Despite the lack of a significant effect of the applied synbiotics on the length and weight of the analysed sections of the small intestine on the 1^st^ day of life, on the 42^nd^ day of rearing, the length of the ileum (*P* ≤ 0.05) was significantly greater in both treatment groups compared to the Control group (Table 4). In both experimental groups, a deepening crypt depth was observed (*P* ≤ 0.01) (Table 7). Other parameters, such as villus height, villus width and villus surface area, were similar in all the groups.

**Table 7:** Effect of synbiotics injected in ovo on the ileum morphology of chickens at 1^st^ and 42^nd^ d of age

Item	Day	
	1	42
Villus height, μm		
Control	295.04 ± 9.16	933.31 ± 43.10
SYN1	298.67 ± 10.86	847.78 ± 50.01
SYN2	289.81 ± 9.09	949.05 ± 38.70
Villus width, μm		
Control	46.42 ± 1.29^b^	162.69 ± 5.58
SYN1	46.62 ± 1.34^b^	166.18 ± 4.45
SYN2	50.85 ± 1.66^a^	161.32 ± 5.30
Villus surface area, μm^2^		
Control	43,547.4 ± 2124.7	471,197.5 ± 20,933.6
SYN1	44,138.3 ± 2318.3	445,293.0 ± 27,184.3
SYN2	45,834.9 ± 1735.9	484,522.9 ± 26,619.7
Crypt depth, μm		
Control	46.14 ± 0.77^B^	91.28 ± 2.59^B^
SYN1	38.55 ± 0.85^C^	116.81 ± 3.18^A^
SYN2	50.14 ± 0.98^A^	117.39 ± 5.53^A^
No. of neutral goblet cells/1mm^2^		
Control	974 ± 50.59^b^	263 ± 26.05
SYN1	1129 ± 61.42^a^	358 ± 40.66
SYN2	678 ± 19.60^c^	285 ± 37.21

^a-c^Difference (*P* ≤ 0.05), ^A-C^difference (*P* ≤ 0.01) between treatments (vertical), Means ± SEM representing 15 birds. SYN 1 - *L. salivarius* IBB3154 + Bi^2^tos, Clasado Ltd., SYN 2 - *L. plantarum* IBB3036 + lupin RFOs

## Discussion

Studying the impact of injected synbiotics on the body weight of chickens throughout their rearing, we found a positive effect of synbiotic 2 on this parameter just in 1-day-old chickens. Opposite, in the research conducted by Bogucka et al. [11], no significant effect of any bioactive substance injected in ovo on the 12^th^ day of incubation on the body weight of 1-day-old chickens was shown.

Our study showed a differential effect of the applied synbiotics (synbiotic 1 - *L. salivarius* IBB3154 + galactooligosaccharides and synbiotic 2 - *L. plantarum* IBB3036 + Raffinose Family Oligosaccharides) on the microstructure of individual sections of a broiler chicken small intestine. We found a positive effect for both synbiotics given in ovo on the height, width and the absorbent surface of duodenum villi in comparison to the Control group on the 1^st^ day of life of the chickens. In turn, on the 42^nd^ day of the life of the chickens, only synbiotic 1 demonstrated a positive impact in comparison to the Control group on the villi width and villi surface area. A similar effect of synbiotics on the width of the villi was observed by Bogucka et al. [12], however, it did not reflect on the absorbent surface of the intestine. Additionally, in birds from the same group at the end of rearing, the deepest crypts were found. The positive effect of in ovo injection of the synbiotic composed of Bi^2^tos and *Lactococcus lactis* subsp. *cremoris* IBB SC1 on the height of duodenum villi on the 1^st^ day of life of chickens was also demonstrated in our previous studies [11]*.* According to Pluske et al. [17], longer villi and their greater absorbent surface area translate into better utilisation of feed, and thereby improve the health of the birds. Deeper crypts, in turn, indicate rapid tissue regeneration processes to permit the renewal of villi to normal sloughing or inflammation due to the presence of pathogens or their toxins [18]. Awad et al. [19], studying the effect of synbiotic supplementation, which is a combination of *Enterococcus faecium* probiotic and a prebiotic derived from chicory rich in inulin and immunomodulatory substances derived from sea algae, did not demonstrate significantly higher villi and significantly deeper crypts in the duodenum of 35-day-old broiler chickens. Similar results were obtained by Awad et al. in their further study in 2009 [20].

In the jejunum of 1-day-old chickens, a beneficial impact of synbiotic 1 on the microstructure was demonstrated, but this wasn’t maintained for 42 d. Similar results in relation to the heights of villi after in ovo administration of bioactive substances (prebiotic: inulin, synbiotics: inulin + *Lactococcus lactis* spp. *lactis,* Bi^2^tos + *Lactococcus lactis spp. cremoris*) in 1-day-old chickens were obtained by Bogucka et al. [11]. At the end of rearing in the group, in which was applied synbiotic 2, significantly higher intestinal villi were stated in comparing to the SYN1 group and the biggest surface area of these villi (in this case no significant differences) which may have an impact on better absorption of nutrients. In our previous studies [12], there were no significant effects of both prebiotics and synbiotics on the height of intestinal villi in 35-day-old broiler chickens.

Analysing the crypts depth of the jejunum in examined groups of birds their significant shortening was stated in both experimental groups (SYN1 and SYN2). The crypt depth is one of the indicators of the health and functional status of the intestine in chickens, and their size can be a measure of the intensity of intestinal epithelial cell renewal processes [21, 22]. Xu et al. [23] indicate that “The crypt can be regarded as the villus factory, and a large crypt indicates fast tissue turnover and a high demand for new tissue” so in our study the decreased crypt depth may indicates an efficient tissue turnover and good condition of the gut. Different results were obtained after in ovo injection of a synbiotic containing the prebiotics inulin and Bi^2^tos. Bioactives had a significant impact on the deepening intestinal crypts of 35-day-old broiler chickens [12]. Rehman et al. [18] examining the effect of the bioactive substance - inulin - on the jejunum histomorphology on the 35^th^ day of the rearing of chickens and found significantly longer villi (*P* ≤ 0.05) and significantly deeper crypts (*P* ≤ 0.01) as a result of this prebiotic supplementation compared to the Control group.

The ileum is characterised by much shorter villi and less absorbent surface of these structures compared to the duodenum and jejunum, which may explain the reduction of the absorption of nutrients at this stage. Evidence of this was the fact that most of the digested food substances had already been absorbed in the upper parts of the small intestine, i.e. the duodenum and jejunum [24]. Injection of the applied synbiotics slightly affected the microstructure of ileum differently compared to the duodenum and jejunum. In 1-day-old chickens, synbiotic *L. plantarum* IBB3036 + lupin RFOs (SYN2 group) significantly increased villi width and crypt depth relative to the other groups. In the studies of Bogucka et al. [11], a synbiotic containing prebiotic Bi^2^tos significantly contributed to the shortening of the villi in 1-day-old chicks, whereas on the 42^nd^ day of rearing, the width of the villi was already significantly lower in both experimental groups than in the Control group. In this section, at the end of rearing, a reduction in the absorbent surface of the villi was found in the SYN1 group in comparison to the Control group while prolonging the crypt depth in both experimental groups. A reduction of the absorbent surface area along with a deepening of the crypts was observed in the group of 35-day-old chickens treated in ovo with a synbiotic containing prebiotic Bi^2^tos and probiotic bacteria *Lactococcus lactis* spp. *Cremoris* in the study of Bogucka et al. [12]. Different results were obtained by Awad et al. [19, 20], which showed a significant extension of the villi and decreased crypt depth in the ileum after application of the synbiotic, although the substance was administered as a feed additive.

The mucus layer in the small intestine is secreted by goblet cells, which permits the excretion of gastric contents and form a protective barrier against mechanical and chemical injuries (ingested food, microorganisms, pathogens) [25, 26]. The percentage of goblet cells in the duodenum is the lowest and increases towards the large intestine throughout the rearing period. This is because a greater number of microbial organisms is present in the proximal colon [27]. This was also confirmed by our findings. On the 1^st^ day after hatching - in groups injected with synbiotics – a significantly lower number of neutral goblet cells on the surface of 1 mm^2^ of duodenum villi was found. At the end of rearing, in the SYN1 group, there were significantly more neutral goblet cells in the jejunum compared to the other groups. In our previous studies [12], the significant effect of the symbiotic (galactooligosaccharides *+ L. lactis* subsp. *cremoris* 477) on an increase in the number of goblet cells in the same segment of the intestine was also shown. However, only a several-fold increase of goblet cells may indicate an infection of the intestinal pathogens [28]. According to Langhout et al. [29] and Sharma et al. [30], the effect for increasing the number of neutral goblet cells both in the jejunum and in the ileum may be due to delayed feed intake by the animals. This results in a reduction in the surface of intestinal absorption and an increase in the number of these cells, which was also confirmed in our results.

The above mentioned research and the results of our study present the different effects of synbiotics, depending on their composition and depending on the anatomical structure of GI tract. These two synbiotics: synbiotic 1 (*L. salivarius* IBB3154 + Bi^2^tos, Clasado Ltd.) and synbiotic 2 (*L*. *plantarum* IBB3036 + lupin RFOs) designed based on our in vitro results [16] presented the two types of synergism, i.e., between the prebiotic and probiotic components (synbiotic 1) and synergism between the two independent bioactive compounds and the host (synbiotic 2). In this last case RFO is less efficiently used by the probiotic bacteria, and it remains available to other indigenous stains of intestinal microbiota. In this situation, the prebiotic has a positive influence on the host organism through improvement of microbial balance in the intestines.

However, the positive effect of injected synbiotics was mainly indicated in the duodenum and jejunum.

## Conclusions/implications

Summarising our research focused on the evaluation of the impact of synbiotics given in ovo on the production traits and histomorphology of a broiler chicken small intestine, we observed that synbiotic *L. salivarius* IBB3154 + Bi^2^tos, Clasado Ltd. and synbiotic *L. plantarum* IBB3036 + lupin RFOs beneficially affected the examined characteristics on the 1^st^ and 42^nd^ day of life. The obtained results allow us to conclude that the use of synbiotics significantly affect gut structure which should contribute to improvement in nutrient absorption by the gut.

## Abbreviations

FCR: Feed conversion ratio
PAS: Periodic acid–Schiff
RFO: Raffinose family of oligosaccharides
SEM: The standard error of the mean
